# Care of HIV-exposed and HIV-infected neonates

**DOI:** 10.4102/sajhivmed.v16i1.360

**Published:** 2015-04-28

**Authors:** Brian Eley

**Affiliations:** 1 Paediatric Infectious Diseases Unit, Red Cross War Memorial Children's Hospital, South Africa; 2 Department of Paediatrics and Child Health, University of Cape Town, South Africa

South Africa has implemented a successful prevention-of-mother-to-child-transmission (PMTCT) intervention programme. Important milestones achieved in 2012 include: (1) an estimated 83% of all pregnant women living with HIV in South Africa received antiretrovirals (ARVs) for PMTCT, (2) early vertical transmission of HIV in infants ≤ 2 months of age declined to 2.4% and (3) early infant HIV diagnosis coverage (i.e. coverage in infants < 2 months of age) reached 72.6%.^1,2^

Complete elimination of mother-to-child transmission (MTCT) will be hard to accomplish. However, further reduction in MTCT may be possible if newborns at high risk of acquiring HIV infection (high-risk infants) after intrapartum exposure are routinely identified and administered an intensified post-exposure prophylaxis (PEP) regimen comprising 2 or 3 ARVs.^3^ The recently updated hospital-level Standard Treatment Guidelines and Essential Medicines List, and the 2014 National Consolidated HIV Guidelines, include risk stratification for newborns.^4,5^ The latter document recommends one of several neonatal PEP regimens depending on the risk of MTCT: (1) for low-risk infants whose mothers were on lifelong antiretroviral therapy (ART) during pregnancy, daily nevirapine (NVP) for 6 weeks is recommended, (2) for high-risk infants because their mothers were on ART for < 4 weeks prior to delivery, or because their mothers were diagnosed with HIV infection within 72 h of delivery, or because their mothers tested HIV positive > 72 h post-delivery, daily NVP for 12 weeks is recommended if breastfed, (3) for infants of breastfeeding mothers with newly diagnosed HIV infection, dual NVP/zidovudine (AZT) prophylaxis is recommended with revision based on the infant's immediate HIV DNA polymerase chain reaction (PCR) result and (4) for high-risk infants born to mothers whose latest viral load results were > 1000 copies/mL, dual NVP/AZT prophylaxis is recommended.^5^ These recommendations may be difficult for the average clinician to digest and remember. Consequently, the extent to which they are correctly administered in routine clinical practice requires evaluation. If implementation proves difficult, a simplified intervention for high-risk infants should be devised.

Identifying high-risk infants and initiating ART when needed is critical to reduce the associated HIV-related morbidity and mortality. A South African study that analysed post-neonatal deaths under 12 months of age identified a peak in mortality between 1 and 3 months owing to HIV infection.^6^ Early survival was estimated in an analysis of pooled individual data from antiretroviral-naïve infants who had been enrolled in 12 sub-Saharan African studies. According to this analysis, net survival of perinatally infected infants declined from 99% at 28 days to 83% by 90 days of life, confirming that a significant mortality risk exists between 1 and 3 months of life.^7^ The Children with HIV Early Antiretroviral Therapy (CHER) trial drew further attention to the vulnerability of HIV-infected infants, convincingly demonstrating that early ART significantly lowered the mortality and disease progression risks of HIV-infected infants. The CHER trial randomly assigned HIV-infected infants aged between 6 and 12 weeks with mild HIV disease (CD4 percentage ≥ 25% and CDC stage N or A disease) to receive ART either immediately (early ART cohort) or when the CD4% had declined to less than 20% (deferred ART cohort). The median age at which ART was initiated was 7.4 weeks in the early ART cohort, and 20 weeks in the deferred ART cohort.^8,9^ Of 532 infants who were screened for eligibility for the CHER trial, 122 (22.9%) were excluded from the trial because they had a CD4 percentage < 25%, were symptomatic or had CDC stage C disease, implying that a sizeable proportion of young HIV-infected infants have advanced HIV disease.^8^ A more recent analysis of 403 infants commenced on ART before the age of 12 weeks in public sector clinics in Cape Town and Soweto showed that, at ART initiation, 250 (62%) had advanced HIV disease.^10^

In June 2008, the World Health Organization (WHO) acknowledged the public health importance of the CHER trial's findings by recommending that, upon diagnosis, all HIV-infected infants (< 12 months of age) should commence ART as soon as possible, irrespective of their clinical stage or CD4 count.^11^ South Africa was slow to embrace this recommendation; on 01 December 2009, 18 months after the WHO report, President Zuma announced in his World AIDS Day speech that the country would implement this intervention.^12^

Although the benefits of early treatment of HIV-infected infants are now well established, identifying these infants remains a challenge. South Africa's practice of routine HIV DNA PCR testing at 6 weeks of life for HIV-exposed infants fails to recognise HIV infection during the first 6 weeks of life, which may be too late to initiate ART at 7.4 weeks of age as per the CHER trial and has reduced sensitivity for detecting HIV infection in infants who receive a minimum of 6 weeks of ARVs for PMTCT.^3^ Birth HIV DNA PCR testing of HIV-exposed neonates coupled with early ART initiation in HIV-infected neonates should be liberalised so as to reduce the observed increase in mortality between 1 and 3 months of age in untreated infants. In 2013, South Africa recommended birth HIV DNA PCR testing for HIV-exposed low birth weight infants, and the 2014 National Consolidated HIV Guidelines recently extended birth testing to 6 categories of newborns deemed to be at high risk of antenatal or intrapartum HIV infection.^4,5^ It will be important to assess how well this recommendation is implemented, and whether it results in increased ART initiation during the neonatal period and reduced HIV-related infant morbidity and mortality.

Finally, recent studies suggesting additional benefits of early ART provide further motivation to identify and treat during the early neonatal period. When ART is initiated in infants less than 6 months of age, the period of viraemia is shortened and the size of the resting CD4+ T-cell latent HIV-1 reservoir is limited.^13^ Continuous decay of this reservoir occurs, provided that virological control is sustained, suggesting that lifelong ART may not be necessary for all patients.^14^ The clinical course experienced by the Mississippi baby suggested that ART initiation within 30 h of life may prolong the control of viral replication in the absence of ART.^15^ However, this supposition was recently challenged by yet another case study. In that report, an HIV-infected neonate was initiated on ART within 30 min of birth, seroreverted and remained clinically well and virologically suppressed while on ART. At the age of 4 years, both HIV-1 RNA and DNA were undetectable. A few weeks later, ART was discontinued, on the assumption that functional cure had been achieved. However, evidence of viral rebound was detected within 7 days of ART discontinuation, raising concerns about the durability of virological control following early neonatal ART.^16^

The challenge for clinicians is the lack of pharmacokinetic and dosing information in neonates for some commonly used ARVs such as abacavir; safety concerns about lopinavir/ritonavir co-formulation in neonates < 14 days of age or infants less than a corrected gestational age of 42 weeks; and uncertainty about the optimal approach of transitioning a neonate from antiretroviral prophylaxis to ART.^17,18^ Because of these limitations, a NVP-containing ART regimen should be administered during the neonatal period until it is safe to prescribe lopinavir/ritonavir co-formulation.^15,16,18^

South African clinicians are grappling with the prevention, diagnosis and management of HIV infection in neonates and a limited body of evidence to guide them. In February 2014, the Southern African HIV Clinicians Society convened a colloquium to review the state of knowledge regarding post-exposure prophylaxis for neonates who experienced high-risk HIV exposures during the intrapartum period, and HIV diagnosis and ART during the neonatal period. The meeting was attended by a group of South African paediatric HIV and AIDS experts (Appendix 1). Arising from this meeting are papers by Max Kroon, Gayle Sherman and James Nuttall addressing these very issues, published in this edition of the *Southern African Journal of HIV Medicine*. These papers provide comprehensive guidance for practising clinicians. The colloquium identified potential research questions, discussed in a separate paper by Mary-Ann Davies.

## Appendix 1

The following individuals, listed in alphabetical order, participated in the colloquium entitled ‘Diagnosis and Treatment of HIV Infection in Neonates’ which took place on 21 February 2014 and/or commented on the series of neonatal papers published in this edition of the *Southern African Journal of HIV Medicine*:

Theunis Avenant
Mo Archary
Ashraf Coovadia
Mark Cotton
Vivian Cox
Mary-Ann Davies
Nonhlanhla Dlamini
Nicolette du Plessis
Brian Eley
Lee Fairlee
Ute Feucht
Lisa Frigati
Ute Hallbauer
Sandy Holgate
Max Kroon
Louise Kuhn
Leon Levin
Aurelie Nelson
James Nuttall
Helena Rabie
Gary Reubenson
Gayle Sherman
Gillian Sorour
Karl Technau
Lloyd Tooke
Kerry Uebel

## References

[1] United Nations Children's Fund Towards an AIDS-free generation – children and AIDS: Sixth stocktaking report. c2013 [cited 03 December 2013]. Available from: http://www.childrenandaids.org/files/str6_full_report_29-11-2013.pdf

[2] Sherman GG, Lilian RR, Bhardwaj S, Candy S, Barron P. Laboratory information system data demonstrate successful implementation of the prevention of mother-to-child transmission programme in South Africa. S Afr Med J. 2014;104(3 Suppl 1):235–238. http://dx.doi.org/10.7196/samj.7598 24893499 10.7196/samj.7598

[3] Nielsen-Saines K, Watts DH, Veloso VG, et al Three postpartum antiretroviral regimens to prevent intrapartum HIV infection. New Engl J Med. 2012;366:2368–2379. http://dx.doi.org/10.1056/NEJMoa1108275 22716975 10.1056/NEJMoa1108275 PMC3590113

[4] National Department of Health Standard treatment guidelines and essential medicines list for South Africa: Hospital level paediatrics. 2013 ed Pretoria: National Department of Health; 2013.

[5] Department of Health National consolidated guidelines for the prevention of mother-to-child transmission of HIV (PMTCT) and the management of HIV in children, adolescents and adults. 24 December 2014. c2014 [cited 10 January 2015]. Available from: http://www.health.gov.za/docs/Policies/2014/HIV_Guidelines_Jan2015-final_edits-YP.pdf

[6] Bourne DE, Thompson M, Brody LL, et al Emergence of a peak in early infant mortality due to HIV/AIDS in South Africa. AIDS. 2009;23:101–106. http://dx.doi.org/10.1097/QAD.0b013e32831c54bd 19065753 10.1097/qad.0b013e32831c54bd

[7] Marston M, Becquet R, Zaba B, et al Net survival of perinatally and postnatally HIV-infected children: A pooled analysis of individual data from sub-Saharan Africa. Int J Epidemiol. 2011;40:385–396. http://dx.doi.org/10.1093/ije/dyq255 21247884 10.1093/ije/dyq255 PMC3140269

[8] Violari A, Cotton MF, Gibb DM, et al Early antiretroviral therapy and mortality among HIV-infected infants. N Engl J Med. 2008;359:2233–2244. http://dx.doi.org/10.1056/NEJMoa0800971 19020325 10.1056/NEJMoa0800971 PMC2950021

[9] Cotton MF, Violari A, Otwombe K, et al Early time-limited antiretroviral therapy versus deferred therapy in South African infants infected with HIV: Results from the children with HIV early antiretroviral (CHER) randomised trial. Lancet. 2013;382:1555–1563. http://dx.doi.org/10.1016/S0140-6736(13)61409-9 24209829 10.1016/S0140-6736(13)61409-9 PMC4104982

[10] Innes S, Lazarus E, Otwombe K, et al Early severe HIV disease precedes early antiretroviral therapy in infants: Are we too late? J Int AIDS Soc. 2014;17:18914 http://dx.doi.org/10.7448/IAS.17.1.18914 24925044 10.7448/IAS.17.1.18914 PMC4056161

[11] World Health Organization Report of the WHO technical reference group, paediatric HIV/ART care guideline group meeting 10–11 April 2008. c2008 [cited 01 July 2008]. Available from: http://www.who.int/hiv/pub/paediatric/WHO_Paediatric_ART_guideline_rev_mreport_2008.pdf?ua=1

[12] UNAIDS President Zuma and UNAIDS executive director, call for mass prevention movement at World AIDS Day commemoration in Pretoria. c2014 [cited 03 November 2014]. Available from: http://www.unaids.org/en/resources/presscentre/featurestories/2009/December/20091201wadms/

[13] Persaud D, Palumbo PE, Ziemniak C, et al Dynamics of the resting CD4(+) T-cell latent HIV reservoir in infants initiating HAART less than 6 months of age. AIDS. 2012;26:1483–1490. http://dx.doi.org/10.1097/QAD.0b013e3283553638 22555165 10.1097/QAD.0b013e3283553638 PMC3495308

[14] Luzuriaga K, Tabak B, Garber M, et al HIV type 1 (HIV-1) proviral reservoirs decay continuously under sustained virologic control in HIV-1-infected children who received early treatment. J Infect Dis. 2014;210:1529–1538. http://dx.doi.org/10.1093/infdis/jiu297 24850788 10.1093/infdis/jiu297 PMC4215073

[15] Persaud D, Gay H, Ziemniak C, et al Absence of detectable HIV-1 viremia after treatment cessation in an infant. N Engl J Med. 2013;369:1828–1835. http://dx.doi.org/10.1056/NEJMoa1302976 24152233 10.1056/NEJMoa1302976 PMC3954754

[16] Butler KM, Gavin P, Coughlan S, et al Rapid viral rebound after 4 years of suppressive therapy in a seronegative HIV-1 infected infant treated from birth. Pediatr Infect Dis J. 2014 9 23 [Epub ahead of print]. 10.1097/INF.0000000000000570 25742088

[17] Panel on antiretroviral therapy and medical management of HIV-infected children Guidelines for the use of antiretroviral agents in pediatric HIV infection. c2015 [cited 08 January 2015]. Available from: http://aidsinfo.nih.gov/contentfiles/lvguidelines/pediatricguidelines.pdf

[18] Food and Drug Administration FDA drug safety communication: Serious health problems seen in premature babies given Kaletra (lopinavir/ritonavir) oral solution. 08 March 2011. c2015 [cited 08 January 2015]. Available from: http://www.fda.gov/drugs/drugsafety/ucm246002.htm

